# Nasal carriage rate and antibiotic susceptibility pattern of *Neisseria meningitidis* in healthy Ethiopian children and adolescents: A cross-sectional study

**DOI:** 10.1371/journal.pone.0187207

**Published:** 2017-10-26

**Authors:** Tinsae Alemayehu, Amha Mekasha, Tamrat Abebe

**Affiliations:** 1 Department of Pediatrics and child health, College of health sciences, Addis Ababa University, Addis Ababa, Ethiopia; 2 Department of Microbiology, Immunology and Parasitology, College of health sciences, Addis Ababa University, Addis Ababa, Ethiopia; University of Cambridge, UNITED KINGDOM

## Abstract

**Background:**

Community nasal meningococcal carriage rates are high across Africa. Meningococcal infections are major causes of morbidity and mortality in the continent; especially among children and adolescents. This study aimed to determine the prevalence of nasal carriage and antibiotic susceptibilities of meningococcal isolates from healthy Ethiopian children and adolescents.

**Method:**

A cross-sectional study was conducted in one of the sub-cities of Addis Ababa, Ethiopia. Nasal swabs were collected and processed for identification, serogrouping and testing susceptibilities for three antibiotics using standard microbiological techniques. Data on epidemiologic risk factors were collected using a structured questionnaire and the magnitude of their association with carriage was assessed using bivariate and multivariate analysis.

**Result:**

A total of 240 samples were collected (115 from males and 125 from females). The mean age of study participants was 11.1 years. The prevalence of nasal carriage for *Neisseria meningitidis* was 20.4% (49/240). Carriage was significantly higher among children living under crowded conditions (OR 1.268; 95% CI: 1.186–1.355; p = 0.006). The predominant serogroups were W135–20/49 isolates (40.8%) and C—12/49 isolates (24.5%) and 83.7% of meningococci were sensitive for Ciprofloxacin. In contrast, isolates showed high resistance to Ceftriaxone (69.4%) while only 4.2% were sensitive for Penicillin. Multi-drug resistance was documented for 14.3% of the isolates.

**Conclusions:**

Meningococcal carriage rate was found to be high with higher rates associated with children and adolescents living in crowded living conditions. Predominant isolates were of serogroup W135 and C and the isolates showed marked susceptibility to Ciprofloxacin and resistance to Ceftriaxone and Penicillin.

## Introduction

*Neisseria meningitidis* is a potentially pathogenic bacteria residing in the nasal cavity. Though carriers are usually asymptomatic, invasive disease can result following its introduction into the blood. Community nasal carriage rates are widely different across the world, ranging from 3%–25%. Meningococcal diseases are responsible for significant morbidity and mortality among children and adolescents. Infections occur at a rate of 500,000 to 1.2 million people per annum; killing 50,000–135,000 of those infected. Meningitis and septicemia are the commonest causes of infections [1]. Thirteen meningococcal serogroups are identified with five (A, B, C, W135 and Y) responsible for almost all invasive illnesses [2].

Carriage rates from the developed world are markedly lower than developing countries. The majority of reports from Europe and North America note rates of 4% or less among healthy children and adolescents [3–5]. Penicillin resistance rates of close to 25% were observed among isolates from children and adolescents in Greece and Turkey [4,6,7]. The samples from these studies were taken from the nasopharynx (Bogaert *et* al reporting on Dutch children and Monteros *et al* from Mexican children and adolescents) [3,5] and the oropharynx (Pavlopulous *et al* who studied carriage in Greek children, Bakir *et al* and Gazi *et al* reporting on colonization among Turkish children [4,6,7]. Reports on predominant serogroups are various for different regions: serotype Y (Mexico and Turkey), C (Greece and Turkey), and W135 (West Africa) [4–9].

Middle-eastern and African studies have revealed significantly higher oropharyngeal meningococcal carriage rates among children and adolescents, ranging from 6.2 to 35%. Carriages were also found to be higher among those aged 10 years or less [8–10]. Studies from the African Meningitis Belt, which spans across more than 20 African countries from Senegal to Ethiopia, report on carriage rates of up to 35% [9–11]. Recent reports indicate that this trend is reversing. The MenAfrCar consortium reported in 2015 that carriage had dropped across seven countries in the African meningitis belt, 5 of whom had introduced the vaccine during the conduct of their study. Overall, pharyngeal carriage rate was 3.4% with ages 5–14 years, males, rural inhabitants and sampling during dry season being significantly related. Serogroup W135 predominated (76.9%). Of the isolates collected from Ethiopia, a 6–7% carriage was noted over the three years the study was conducted [12]. Basta et al also described a carriage prevalence of 3.2% among Ethiopian children in 2013 [11]. A report from Gondar University teaching hospital noted 6% prevalence with all nasopharyngeal isolates being resistant to Co-trimoxazole, half of isolates resistant to Ciprofloxacin and one-fifth resistant to Ceftriaxone [13].

Ethiopian data concerning nasopharyngeal meningococcal carriage, serotypes involved and susceptibility testing for antibiotics conventionally used for treatment and prophylaxis of invasive meningococcal diseases is scarce. The aim of our study was to determine the characteristics of nasal meningococcal carriage as well as antibiotic susceptibilities of isolates among healthy Ethiopian children in Addis Ababa.

## Materials and method

The methodology and laboratory protocol can be cited at dx.doi.org/10.17504/protocols.io.jfbcjin

### Study setting and period

The study was conducted in Major General Hayelom Araya Primary and Junior Secondary school, located in the Lideta sub-city (district) of Addis Ababa City Administration. Addis Ababa has an estimated population of 3.6 million living in 10 sub-cities (districts). An estimated 214,769 inhabitants reside in Lideta sub-city. The sub-city is located in the central western part of Addis Abeba and is the second most densely populated of the city’s 10 sub-cities with approximately 23,000 people per square kilometer. Data was collected in the months of March–May, 2017.

### Study design

The study was a cross-sectional study. All children and adolescents enrolled in the study school were offered inclusion in the study with the exception of those with an acute respiratory infection within the preceding seven days those confirming receipt of antibiotics within the same period of time. Those children and adolescents under care for immunodeficiency, hematologic or oncologic disorders, receiving immune-suppressing medications, having a cranio-facial malformation and those whose participation in the study was refused by their parents or guardians were also excluded. Simple random sampling was employed.

### Source population

The sub-city has 16 governmental and 1 public primary and junior secondary schools as well as 3 governmental secondary schools. All children and adolescents enrolled under the governmental and public primary and junior secondary schools were the source population.

### Study population

The Major General Hayelom Araya Primary and Junior Secondary school enrolls 659 students, of which 323 are boys and 336 are girls.

### Sample size

Simple random sampling was utilized in selecting the school from its governmental and public-run counterparts in the sub-city. All children and adolescents were offered participation in the study and in keeping with the inclusion criteria, were recruited till the sample size was attained. A sample size of 240 children and adolescents was calculated citing the prevalence noted by Assefa *et al* from Northwestern Ethiopia: p = 0.06, z = 1.96 [13].

### Data collection methods

Study participants were recruited after a written informed consent was obtained from parents or guardians. Socio-demographic data and information concerning epidemiologic risk factors for meningococcal carriage were collected using a structured and standardized questionnaire prior to sample collection. Crowding within household was defined as the residence of more than one person per room [14].

### Microbiologic procedures

#### A. Sample collection and processing

Nasal swabs were collected by a trained medical microbiologist using pre-moistened sterile cotton swabs. Following insertion of the swab about one inch (2.5 cm) into the nostril, it was rolled five times and reinserted to the second nostril and the sampling steps were repeated. After collection, samples were inserted immediately into Amies media (Beckton Dickinson Co., USA) and were transported to the laboratory within one hour in a cold box.

#### B. Culture and identification

Culture and identification was undertaken by the microbiology laboratories of Tikur Anbessa Specialized Hospital, Addis Ababa. The agar medium was Mueller Hinton Agar with sheep blood and was produced in-house. Sample was inoculated on sheep blood agar and chocolate agar by rolling and streaking of swab over plates using a sterile loop. Inoculated plates were incubated at 37°C under a 5% CO_2_ enriched atmosphere for 72 hours.

Plates were examined for growth of typical colonies every 24 hours. After oxidase positivity was confirmed, gram staining was done.

After confirming for the presence of gram negative diplococci, isolates were sub-cultured on sheep blood or chocolate agar to guarantee purity of colonies. Following this, carbohydrate utilization tests (glucose, maltose, lactose and sucrose) were used to further confirm colonies. Gram negative, oxidase positive, glucose positive, maltose positive, lactose negative and sucrose negative diplococci were confirmed as *Neisseria meningitidis*. A quality strain of ATCC13090 was used as a control.

Serogrouping of isolates was done after mixing colonies with normal saline on a slide to confirm purity by observing for absence of precipitates. Then slide agglutination method was employed using mixtures of colonies with standard antisera for the different serotypes of *Neisseria meningitidis* (Beckton Dickinson Co., USA). The presence of precipitates during mixing was used to check for the specific serotypes. Serogrouping was performed against antisera for serogroups A, B, C, W135, X, Y and Z. If negative for these seven serogroups, it was classified as non-serogroupable. We could not access further selective tests including polymerase chain reaction (PCR) tests and molecular characterization in our laboratories.

#### C. Antibiotic susceptibility testing

Antibiotic susceptibility testing was done using disc diffusion method. A standardized 0.5 McFarland tryptometer was used to confirm inoculum density. Colonies were coated on Muller Hinton and sheep blood agars and inoculated at 37°C under a 5% CO_2_ enriched atmosphere for 24 hours. Susceptibility to antibiotics was confirmed according to the Clinical and Laboratory Standard Institute (CLSI) and the British Society of Anti-microbial Chemotherapy guidelines. Antibiotic disks for Ceftriaxone, Ciprofloxacin and Penicillin were used (Beckton Dickinson Co., USA), with concentrations of 30 μg, 5 μg and 10 μg, respectively.Susceptibility to Ceftriaxone was taken as a zone diameter of greater than or equal to 34 mm while resistance was defined as less than 34 mm. Ciprofloxacin susceptibility was defined as a zone diameter of greater than or equal to 35 mm; intermediate resistance as 33–34 mm and resistance as less than or equal to 32 mm. Penicillin sensitivity was taken for zone diameters of more than 28 mm; intermediate resistance as 15–28 mm and resistance as less than or equal to 14 mm [15–17].

Multi-drug resistance was defined as resistance of an isolate to two or more of the antibiotics tested. Isolates were tested against Ceftriaxone, Ciprofloxacin and Penicillin. These antibiotics were chosen because they are standard recommendations for treatment and prophylaxis against meningococcal diseases. Testing of resistant isolates for β-lactamase was not possible.

### Statistical analysis

All recorded data were transferred to SPSS version 23.0. Data were summarized using descriptive statistics, frequencies, and tables. The magnitude of association between the different variables and nasal meningococcal carriage was assessed using bivariate and multivariate analysis and expressed in odds ratio and 95% confidence interval. Statistically significant differences were taken at p < 0.05.

### Ethical considerations

The study protocol was approved by the Research and Publications Committee of the Department of Pediatrics and Child health, College of health sciences, Addis Ababa University. Permission to undergo the study was also granted by the Education bureau of Lideta sub-city administration, Addis Ababa.

## Results

Overall, 240 children and adolescents (115 males and 125 females) were included in the study. Their mean age was 11.1 years. A majority (84.3%) lived in Lideta sub-city whereas the rest resided in eight of the other nine sub-cities of the city administration. The average family size was 5–6 people per household. Average number of rooms per household is two. Crowded living conditions were the predominant observation (96.7%) with approximately 57% of families earning less than 1 US dollar and 25 cents per day (extreme poverty). Charcoal and electricity were the preferred tools for cooking, among 55.8% and 30.4% of respondents respectively. Parental cigarette smoking was stated by 16.2% of participants while 58.3% were reported to be present in areas of mass gatherings in the recent past (Table 1).

**Table 1:** Socio-demographic and clinical characteristics of all participants.

Characteristics of children (n = 240)		n	%
Gender distribution	Male	115	47.9%
	Female	125	52.1%
Age distribution	Younger than 7 years	2	0.8%
	7–10 years	116	48.3%
	11–14 years	97	40.5%
	Older than 14 years	25	10.4%
Residential sub-city	Addis Ketema	14	5.8%
	Akaki Kality	1	0.4%
	Arada	12	5.0%
	Bole	5	2.1%
	Kirkos	2	0.8%
	Kolfe Keranio	1	0.4%
	Lideta	202	84.3%
	Nifas Silk Lafto	1	0.4%
	Yeka	2	0.8%
Religion of family	Orthodox	176	73.3%
	Moslem	47	19.6%
	Protestant	15	6.3%
	Catholic	2	0.8%
Housing conditions	Crowded	232	96.7%
	Not crowded	8	3.3%
Family income	Less than 1.25 USD per day	137	57.1%
	1.25–2.50 USD per day	41	17.1%
	2.50–5.50 USD per day	33	13.7%
	5.50–8.00 USD per day	11	4.6%
	More than 8.00 USD per day	18	7.5%
Cooking practices	Indoor cooking	192	80.0%
	Outdoor cooking	48	20.0%
Cooking tools	Cow dung (*Kubet)*	3	1.3%
	Dry wood	5	2.1%
	Charcoal (*Kesel*)	134	55.8%
	Gas	25	10.4%
	Electricity	73	30.4%
Parental cigarette smoking	Yes	39	16.2%
	No	201	83.8%
Presence in mass gatherings	Yes	140	58.3%
	No	100	41.7%

The mean classroom size was 42.5 students. This adheres to the recommendations for a standard, adequately spaced classroom (40 students per classroom) of the Education Bureau of the City Administration. As meningococcal vaccines are not offered in the national program of immunization, none of the study participants received any immunizations against *Neisseria meningitdis*.

*Neisseria meningitidis* was isolated from 49 of 240 nasal swabs (20.4%). Crowded living conditions were seen to be significantly associated with carriage after bivariate and multivariate analysis (Tables 2 and 3).

**Table 2:** Characteristics of the carrier population and characteristics of isolates.

			Characteristics of isolates (n = 49)							
Characteristics of meningococcal carriers (n = 49)		Total	A (n = 6)	B (n = 2)	C (n = 12)	W135 (n = 20)	X (n = 1)	Y (n = 3)	Z (n = 0)	NS (n = 5)
Gender distribution	Male	29	3		10	10	1	3		2
	Female	20	3	2	2	10				3
Age distribution (in years)	< 7	1						1		
	7–10	29	3	1	9	10	1	2		3
	11–14	19	3	1	3	10				2
	> 14	0								
Residential sub-city	Addis Ketema	4			1	3				
	Arada	1			1					
	Bole	1			1					
	Kirkos	1						1		
	Lideta	41	6	2	9	16	1	2		5
	Yeka	1				1				
Religion of family	Orthodox	36	5	2	10	12	1	3		3
	Moslem	7	1		1	5				
	Protestant	4				3				1
	Catholic	2			1					1
Housing Conditions	Crowded	49	6	2	12	20	1	3		5
	Not crowded	0								
Daily family Income (USD)	< 1.25	33	1		9	17	1	2		3
	1.25–2.50	6	1	1	2	1		1		
	2.50–5.50	9	4		1	2				2
	5.50–8.00	0								
	> 8.00	1		1						
Cooking practices	Indoor cooking	44	6	1	9	19	1	3		5
	Outdoor cooking	5		1	3	1				
Cooking tools	Cow dung (*Kubet)*	0								
	Dry wood	3	1		2					
	Charcoal (*Kesel*)	28	1		6	16	1	1		3
	Gas	5	1			3		1		
	Electricity	13	3	2	4	1		1		2
Parental cigarette smoking	Yes	7			2	3				2
	No	42	6	2	10	17	1	3		3
Presence in mass gatherings	Yes	30	3		7	14	1	2		3
	No	19	3	2	5	6		1		2

Key: NS–Non-serogroupable

**Table 3:** Bivariate and multivariate analysis of risk factors for carriage among participants.

Variable		*N*. *meningitidis*		Odds ratio with 95% CI	
		No (n = 191)	Yes (n = 49)	Crude OR (95% CI)	P value
Gender	Female	105 (84%)	20 (16%)	0.565 (0.299–1.068)	0.077
	Male	86 (74.8%)	29 (25.2%)		
Age (in years)	Less than 7	1 (50%)	1 (50%)	0.253 (0.016–4.112)	0.297
	7–10	87 (75%)	29 (25%)	0.577 (0.305–1.091)	0.088
	10–14	78 (80.4%)	19 (19.6%)	0.918 (0.482–1.745)	0.793
	More than 14	25 (100%)	0		
Residential sub-city	Addis Ketema	10 (71.4%)	4 (28.6%)	2.057 (0.669–6.322)	0.200
	Arada	11 (91.7%)	1 (8.3%)	0.341 (0.043–2.706)	0.287
	Bole	4 (80%)	1 (20%)	0.974 (0.106–8.915)	0.981
	Kirkos	1 (50%)	1 (50%)	0.253 (0.016–4.112)	0.297
	Lideta	161 (79.7%)	41 (20.3%)	0.828 (0.364–1.883)	0.652
	Yeka	1 (50%)	1 (50%)	0.253 (0.016–4.112)	0.297
Religion	Orthodox	140 (79.6%)	36 (20.4%)	0.935 (0.466–1.878)	0.851
	Moslem	40 (85.1%)	7 (14.9%)	1.589 (0.664–3.804)	0.295
	Protestant	11 (73.3%)	4 (26.6%)	0.688 (0.209–2.260)	0.535
	Catholic	0	2 (100%)	0.124 (0.011–1.393)	0.056
Housing conditions	Crowded	183 (78.9%)	49 (21.1%)	1.268 (1.186–1.355)	0.006
	Not crowded	8 (100%)	0		
Daily family income	< 1.25 USD	104 (75.9%)	33 (24.1%)	1.725 (0.890–3.343)	0.104
	1.25–2.5 USD	35 (85.4%)	6 (14.6%)	1.608 (0.635–4.073)	0.313
	2.5–5.5 USD	24 (72.7%)	9 (27.3%)	0.608 (0.262–1.415)	0.245
	5.5–8.0 USD	11 (100%)	0		
	> 8.0 USD	17 (94.4%)	1 (5.6%)	4.690 (0.609–36.136)	0.104
Cooking practices	Indoor cooking	148 (77.1%)	44 (22.9%)	2.557 (0.954–6.849)	0.055
	Outdoor cooking	43 (89.6%)	5 (10.4%)		
Cooking tools	Cow dung (*Kubet)*	3 (100%)	0		
	Dry wood	2 (40%)	3 (60%)	0.245 (0.048–1.252)	0.069
	Charcoal (*Kesel*)	106 (79.1%)	28 (20.9%)	0.935 (0.496–1.763)	0.836
	Gas	20 (80%)	5 (20%)	0.792 (0.298–2.103)	0.639
	Electricity	60 (82.2%)	13 (17.8%)	0.788 (0.390–1.594)	0.507
Parental cigarette smoking	Yes	32 (82.1%)	7 (17.9%)	0.668 (0.263–1.698)	0.394
	No	159 (79.1%)	42 (20.9%)		
Presence in mass gatherings	Yes	110 (78.6%)	30 (21.4%)	0.938 (0.495–1.777)	0.990
	No	81 (81%)	19 (19%)		

The average family size among children with meningococcal nasal carriage was 5 people per household. Average number of rooms per household was 1.9 per household. Predominant serogroups were W135–20/47 isolates (42.6%) and C—11/47 isolates (23.4%). Serogroup A was isolated from 6 participants (12.2%) while the rest 12 meningococci were of serotypes B, X, Y and non-serogroupable variants. Serogroup C was more commonly seen among male children aged 7–10 years living in extreme poverty (Table 3). Carrier isolates were not stored for further confirmation.

Based on standard recommendation on zone diameter measurements, the colonies showed high resistance for Ceftriaxone (69.4%). In contrast, isolates were highly sensitive to Ciprofloxacin (83.7%). Penicillin sensitivity was markedly low (4.2%) while the bulk of meningococci showed intermediate resistance to Penicillin (83.3%) and complete resistance (12.2%). Multi-drug resistance (resistance to two or more of the antibiotics) was documented for 14.3% of isolates (Table 4).

**Table 4:** Antimicrobial susceptibility patterns of meningococcal isolates.

Antimicrobials		*N meningitidis* (n = 49)							
		A (n = 6)	B (n = 2)	C (n = 12)	W135 (n = 20)	X (n = 1)	Y (n = 3)	NS (n = 5)	Total
Ceftriaxone*	Sensitive	2		4	7		1		15
	Resistant	4	2	8	13	1	2	5	34
Ciprofloxacin*	Sensitive	6	2	9	17	1	1	5	41
	Intermediate	0		3	3		1		7
	Resistant	0					1		1
Penicillin^^^	Sensitive	0			2				2
	Intermediate	6	2	10	14	1	3	5	41
	Resistant	0		2	4				6

* 2016 CLSI guidelines (16)

^^^ 2015 BSAC guidelines (15)

## Discussion

This study showed a significant meningococcal nasal carriage (20.4%) among apparently healthy Ethiopian children and adolescents. This was markedly higher than seen among pharyngeal isolates from Ethiopian school children in multi-center African surveillances done between 2010–2013 which showed a prevalence of 3.2–3.4% [11]. It also showed more than three times more carriage rates as compared to the 6% carriage prevalence documented by Assefa *et al* [13] among nasopharyngeal samples collected from children aged 10 years or less visiting Gondar University hospital, Northwestern Ethiopia. Contrary to local studies and those reported from the Netherlands, Mexico (both nasopharyngeal samples) and Greece (oropharyngeal samples) [3–5], its findings stand in line with high rates reported from the western parts of the African meningitis belt during the pre-serogroup A meningococcal vaccine (MenAfriVac) era: Mali (15.5%), Burkina Faso (22.6%) and Niger (35%) as well as from oropharyngeal samples from Iranian children (12.1%) [8,9,11].

Our observations may have been due to the extreme poverty and crowded living conditions seen in the study site. They could also be related to the hot and dry season during which the study was conducted (March–May) which is suitable for meningococcal transmission. It should be noted that the study period did not coincide with the time of the Hajj pilgrimage. Our findings necessitate future researches involving isolates stored for PCR testing and molecular characterization.

A risk factor identified in our study in association with a high carriage was living under crowded conditions. Radiological confirmation of pulmonary tuberculosis was positively associated with nasopharyngeal carriage in Gondar, Ethiopia [13]. Older aged Greek children (age more than 12 years) showed increased carriage as compared to those aged 12 years or less (4). This contrasted to young age being significantly associated with carriage among Dutch children; which also showed youth club visits and pneumococcal carriage being other associated factors [3]. Fahimzad et al noted that *Hemophilus influenzae* nasal carriage and a recent respiratory infection were significant associations with childhood meningococcal carriage in Iran [8].

Poverty and overcrowding were important determinants among Mexican children aged less than 5 years and teenagers [5].

Predominant serogroups in our study site were W135 (40.8%) and C (24.5%). This compares to serogroup Y (Mexico and Turkey), C (Greece and Turkey), and W135 (West Africa) [4–9]. Our findings help in providing local data for use in selection of a meningococcal conjugate vaccine projected for incorporation of the national program of immunization. They could also serve as a baseline for a national carrier screening study in comparison to isolates from clinical invasive disease.

The other notable findings seen in this study were low degrees of sensitivity of isolates for Ceftriaxone and Penicillin as well high susceptibility for Ciprofloxacin. This contrasts with the findings from isolates grown from nasal swabs in Gondar University, north western Ethiopia where only 21.4% were resistant to Ceftriaxone and half were resistant to Ciprofloxacin [13]. The low degree of Penicillin sensitivity echoes what was seen in Greek and Turkish children where Penicillin resistance was documented in close to a quarter of meningococcal isolates [4,7].

These findings may reflect on the injudicious use of commonly available antibiotics which has multiple underlying factors in our society.

In conclusion, meningococcal carriage was found to be high among healthy Ethiopian children and adolescents in Addis Ababa. Nasal carriage was high among children and adolescents living in crowded states. Predominant isolates were of serogroup W135 and C. Meningococci showed marked resistance to Ceftriaxone and Penicillin while Ciprofloxacin was the drug for which most isolates were sensitive. Our findings have implications on the inclusion of meningococcal vaccines into the Ethiopian national program of immunization and its possible effects on the meningococcal population.

## Supporting information

S1 Table Meningococcal carriage.sav. (SAV)
